# Factors associated with knowledge of the disease in people with type 2 diabetes mellitus

**DOI:** 10.17533/udea.iee.v39n1e02

**Published:** 2021-03-05

**Authors:** Verônica Rabelo Santana Amaral, Ícaro José Santos Ribeiro, Roseanne Montargil Rocha

**Affiliations:** 1 . Nurse, Master´s degree. Universidade Estadual de Santa Cruz, Ilhéus, Bahia - Brazil. Email: vekarabelo@gmail.com Universidade Estadual de Santa Cruz Universidade Estadual de Santa Cruz IlhéusBahia Brazil vekarabelo@gmail.com; 2 . Nurse, Ph.D. Universidade Estadual do Sudoeste da Bahia, Jequie, Bahia - Brazil. Email: icaro.ribeiro29@gmail.com Universidade Estadual do Sudoeste da Bahia Universidade Estadual do Sudoeste da Bahia JequieBahia Brazil icaro.ribeiro29@gmail.com; 3 . Nurse, Ph.D. Adjunct Professor. Universidade Estadual de Santa Cruz, Ilhéus, Bahia - Brazil. Email: roseannemontargil@gmail.com Universidade Estadual de Santa Cruz Universidade Estadual de Santa Cruz IlhéusBahia Brazil roseannemontargil@gmail.com

**Keywords:** health education, primary health care, self-management, diabetes mellitus, risk factors, educación en salud, atención primaria de salud, automanejo, diabetes mellitus, factores de riesgo, educação em saúde, atenção primária à saúde, autogestão, diabetes mellitus, fatores de risco

## Abstract

**Objective.:**

To identify factors associated with the level of knowledge of the disease in people with type 2 Diabetes.

**Methods.:**

A cross-sectional study carried out with 412 people with diabetes registered in the Primary Health Care network of a Brazil Northeast municipality. For data collection, we used a questionnaire with sociodemographic and clinical variables and to identify the level of knowledge, we used the *Diabetes Knowledge Questionnaire*.

**Results.:**

Insufficient knowledge prevailed in 54.7% of the participants, associated in significant bivariate analysis (p<0.05) with the sociodemographic variables: age (≥ 60 years old), marital status (without a partner), education (up to complete / incomplete elementary school), family income (≤ 1 minimum wage). For clinical variables, the level of insufficient knowledge was significantly associated with not participating in an educational group, not using insulin, and not practicing physical activity. In logistic regression, we observed that the factors that increase the risk for insufficient knowledge were: never having participated in an educational group (OR=2.0), age ≥ 60 years old (OR=2.2), illiterate and primary education (OR=8.3) and income less than or equal to 1 minimum wage (OR = 2.4).

**Conclusion.:**

The level of knowledge of people with type 2 diabetes mellitus about their disease is insufficient, with socioeconomic and educational characteristics being the factors that increase the odds of having this level of knowledge**.**

## Resumen

**Objetivo.:**

Identificar los factores asociados al nivel de conocimiento de la enfermedad en personas con Diabetes Mellitus Tipo 2.

**Métodos.:**

Estudio transversal realizado con 413 personas con diabetes inscritas en la red de Atención Primaria de Salud de un municipio del Nordeste de Brasil. Para la recolección de los datos, se utilizó un cuestionario con variables sociodemográficas y clínicas y, para identificar el nivel de conocimiento, se utilizó el *Diabetes Knowledge Questionnaire*.

**Resultados.:**

Prevaleció el conocimiento insuficiente en el 54.7% de los participantes, asociándose significativamente en el análisis bivariado (*p*<0.05) con las variables sociodemográficas: edad (≥ 60 años), estado civil (sin pareja), escolaridad (hasta primaria completa / incompleta) e ingreso familiar (≤1 salario mínimo). En cuanto a las variables clínicas, el nivel de conocimiento insuficiente se asoció significativamente con no participar en un grupo educativo, no usar insulina y no practicar actividad física. En la regresión logística, se observó que los factores que incrementan el riesgo de conocimiento insuficiente fueron: nunca haber participado en un grupo educativo (OR=2.0), edad ≥ 60 años (OR=2.2), nivel educativo de analfabetismo o de educación primaria (OR=8.3) e ingresos menores o iguales a 1 salario mínimo (OR=2.4).

**Conclusión.:**

El nivel de conocimiento sobre su enfermedad de las personas con Diabetes Mellitus Tipo 2 es insuficiente, siendo las características socioeconómicas y educativas los factores que más aumentan la probabilidad de tener este nivel de conocimiento.

## Resumo

**Objetivo.:**

Identificar os fatores associados ao nível de conhecimento da doença em pessoas com Diabetes Mellitus tipo 2.

**Métodos.:**

Estudo transversal realizado com 413 pessoas com diabetes cadastradas na rede de Atenção Primária a Saúde de um município do Nordeste do Brasil. Para coleta de dados foi utilizado questionário com variáveis sociodemográficas e clínicas e para identificar o nível de conhecimento foi utilizado o instrumento *Diabetes Knowledge Questionnaire*.

**Resultados.:**

Prevaleceu o nível de conhecimento insuficiente em 54.7% dos participantes, associando-se em uma análise bivariada de forma significativa (p<0.05) com as variáveis sociodemográficas: idade (≥ 60 anos), situação conjugal (sem companheiro), escolaridade (até o ensino fundamental completo/incompleto), renda familiar (≤ 1 salário mínimo). Quanto as variáveis clínicas, o nível de conhecimento insuficiente se associou de forma significativa com a não participação em grupo educativo, não utilização de insulina e não praticar atividade física. Em regressão logística se observou que os fatores que aumentam o risco para o conhecimento insuficientes foram: nunca ter participado de grupo educativo (OR=2.0), idade ≥ 60 anos (OR=2.2), escolaridade analfabeto e primário (OR=8.3) e renda menor ou igual a 1 salário mínimo (OR=2.4).

**Conclusão.:**

O nível de conhecimento das pessoas com diabetes mellitus tipo 2 acerca de sua doença é insuficiente, sendo as características socioeconômicas e educacionais os fatores que aumentam as razões de chances do conhecimento insuficiente.

## Introduction

Diabetes mellitus (DM) is a chronic condition of high prevalence that consists of a metabolic disorder with a deficiency in the production and/or action of insulin, characterized by persistent hyperglycemia, which is the determining factor in the diagnosis, treatment, and prevention of complications. It is considered a public health problem with approximately 425 million cases registered in 2017, with a growth estimate of 629 million adults by 2045.(1) The most common is type 2 diabetes mellitus (DM2), representing 90 to 95% of cases, characterized by a deficiency in insulin action/production and insulin resistance, with advanced age, obesity, lack of physical activity, and family history as the risk factors for its development.(2)

A study carried out by the Surveillance of Risk and Protection Factors for Chronic Diseases by Telephone Survey (VIGITEL, Portuguese initials) in Brazil found that diabetes affects 7.7% of the population and the frequency of this condition increases considerably with advancing age and decreased with more levels of education.(3) Factors such as socioeconomic and demographic variables (male gender; race/skin color black or brown), lifestyle (smoking; increased waist-hip ratio), individual's health condition (use of insulin; poor self-rated health), functional category compatible with high school (technical position) and not having a health plan are associated with a greater chance of inadequate glycemic control.(4) The therapeutic plan aims at the appropriate glycemic control that needs drug treatment, lifestyle changes, physical activity, and health education to be achieved.(5) As a way of collaborating in the execution of these activities, educational interventions have shown positive results in knowledge about diabetes, adherence to drug treatment, and glycated hemoglobin rates.(6)

Practices based on empowerment through an approach mainly on problematization to acquire knowledge, to obtain essential skills and attitudes to live with diabetes contribute to the achievement of adequate glycemic control.(7) Therefore, health education represents an important pillar in the treatment of diabetes since the care measures are implemented through knowledge of the mechanisms that involve the disease. Thus, the greater the knowledge, the greater the chances of adopting positive attitudes in self-management of health,(8) so nurses must consider the potential of educational tools and use them in the monitoring of patients with DM2. In this context, Primary Health Care (PHC) is the scenario capable of promoting health education programs that improve the self-care indicators of people with diabetes, inserting them in the therapeutic process. PHC is the structuring axis of the Brazilian Unified Health System (SUS), being the first and broadest level of care in the country, responsible for health promotion, prevention, and treatment of diseases such as Diabetes Mellitus.(9) Therefore, during the follow-up of people with diabetes in PHC, we need to assess the level of knowledge about the disease and identify the associated factors, as this will support the educational actions that assist in the treatment of people with DM2. Thus, this study aimed to identify the factors associated with the level of knowledge of people with type 2 Diabetes Mellitus registered in the PHC of a municipality in the Northeast of Brazil.

## Methods

This is a cross-sectional study with a quantitative approach, carried out in a municipality in the Northeast region of Brazil, with an estimated population of 213 685 inhabitants. The municipality has thirty Health Units in its PHC Network, which are geographically divided into four Assistance Modules. This research chose one unit of each Assistance Module for data collection, defined by a proportional sampling of size, totaling four Health Units. Based on the population of people with DM2 registered in the four Health Units of the research (*n*=1115), insufficient knowledge prevalence of 56.1%(10) in people with DM2 and adopting the parameters of 99% confidence level and 1% estimation error, we obtained a necessary sample of 420 individuals. We selected 420 participants by simple random sampling from the list of people with DM2 in each unit, and afterward, we conducted a home visit guided by the Community Health Agent (CHA). The inclusion criteria were: being eighteen years old or older, having a medical diagnosis of DM2, being registered in one of the units where data collection took place, being able to understand and answer questions in the questionnaire and instrument, and agreeing to participate in the research by signing the Free and Informed Consent Term. The exclusion criterion adopted was the absence of answers to the questions of the research instruments. Thus, we excluded 7 participants, totaling 413 individuals for data analysis.

The outcome variable of the study was the knowledge of people with DM2 about the disease. To assess the level of knowledge about diabetes, we used the Diabetes Knowledge Questionnaire (DKN-A) instrument,(11) translated into Portuguese and validated in Brazil and we presented it in the analysis of reliability and test-retest, the Kappa coefficient ranging from 0.56 to 0.69. (12) Also, this instrument is widely used in national and international scientific research, indicated by the Brazilian Diabetes Society. (2) The DKN-A is composed of fifteen multiple-choice questions that address categories related to DM: basic physiology, hypoglycemia, food and substitutions, management, and self-care. For each correct answer, 1 point is counted, with the measurement scale from 0 to 15. Scores less than or equal to 7 are classified as insufficient knowledge about diabetes.

The variables of the sociodemographic conditions were: gender (male and female); marital status (living with and without a partner); age; school level; occupation (with and without occupation); family income; the number of residents in the house. The self-reported clinical variables evaluated were: time of diagnosis DM2 (in years); educational group (participating or participated in an educational group and never participated); treatment (does not use insulin and using insulin); medical consultation and nursing consultation (length of consultation, ≤ 3 months adequate follow-up, and> 3 months inadequate follow-up); performing physical activity (yes and no).

Data collection took place through a home visit that was guided by the CHA, from December 2017 to June 2018. The collection team consisted of six students in the health area, scholarship holders from a state public university in Brazil, duly trained. At home, the objective of the research was presented and when accepted, an invitation to participate was formalized through reading and signing the informed consent form. When denied or absent at the time of the visit, the collection team sought another residence until the quantitative data established by the health unit. We sought to perform data collection in the residence environment that had less noise and interference, started with the application of the sociodemographic and clinical questionnaire followed by the DKN-A instrument.

The data were analyzed using the Statistical Package for the Social Sciences (version 21.0). We performed a descriptive analysis of sociodemographic and clinical variables and calculated the frequencies (absolute and relative). After the non-normality was confirmed by the Shapiro Wilk test, the association of diabetes knowledge and the sociodemographic and clinical characteristics was verified using bivariate analysis, with Pearson's chi-square test. The variables that presented p <0.2 in the bivariate analysis were inserted in a binary logistic regression model, stepwise backward method, with the results expressed in Odds Ratio and 95% confidence interval, with significance at p <0.05 for all analyzes.

This research was approved by the Research Ethics Committee of the State University of Southwest Bahia, under the opinion 2,346,497 of October 24, 2017, through the number of CAAE 74607417.6.0000.0055, following Resolution 466/12 of the National Council of Cheers.

## Results

We interviewed 420 people with DM2. There was a loss of 8 participants who showed no answers in the DKN-A instrument, totaling 412 people for data analysis. Table 1 shows that the most prevalent demographic characteristics in the studio group were: female (69.7%), 60 years old and older (67.7%), without a partner (57.5%), with elementary education (91.2%), not working outside (work activities carried out outside the home environment) (84.5%), with a family income less or equal to a minimum wage (41%) and living with 1 to 3 people.

The level of knowledge of the population with DM2 about their disease was insufficient in 54.7% of the participants. Table 1 shows the characteristics of the population according to the level of knowledge. Most people with insufficient knowledge were female, age ≥ 60 years old, living without a partner, studied up to complete/incomplete elementary school, did not work outside home, and had a family income ≤1 salary and living with 1 to 3 people.

The level of knowledge of the population with DM2 about their disease was insufficient in 54.7% of the participants. Table 1 also presents the characteristics of the population according to the level of knowledge. We found a statistically significant difference for the proportion of insufficient knowledge in the variables: age (≥ 60 years old), marital status (living without a partner), school level (studied up to elementary school), and family income (incomes ≤1 wage).

**Table 1:** Sociodemographic data of the study population according to total and level of knowledge about diabetes mellitus

Variables	Level of Knowledge	Level of Knowledge	*p-value*	Total *n* (%)
Variables	Sufficient *n* (%)	Insufficient *n* (%)	*p-value*	Total *n* (%)
Total	187 (45.3)	226 (54.7)		412 (100.0)
Gender			0.874	
Female	131 (70.1)	156 (69.3)		287 (69.7)
Male	56 (29.9)	69 (30.7)		125 (30.3)
Age			<0.001	
< 60 years old	86 (46)	47 (20.9)		133 (32.3)
≥ 60 years old	101 (54)	178 (79.1)		279 (67.7)
Marital status			0.015	
With a partner	89 (48.1)	80 (36.2)		169 (41.0)
Without a partner	96 (51.9)	141 (63.8)		237 (57.5)
Without information				6 (1.5)
Education level	3 (1.6)	45 (20.2)	<0.001	
Illiterate	40 (21.7)	66 (29.6)		48 (11.7)
Primary	55 (29.9)	81 (36.3)		106 (25.7)
Elementary	62 (33.7)	28 (12.6)		136 (33.0)
High school	24 (13.0)	3 (1.3)		90 (21.8)
Higher education				27 (6.6)
Without information	36 (19.4)	28 (12.4)		5 (1.2)
Occupation	150 (80.6)	198 (87.6)	0.052	
With occupation				64 (15.5)
Without occupation	58 (31.7)	111 (52.1)		348 (84.5)
Family income*	97 (53)	89 (41.8)	<0.001	
≤ 1 minimum wage	28 (15.3)	13 (6.1)		169 (41.0)
2 - 3 minimum wages				186 (45.1)
≥ 4 minimum wages	0 (0)	2 (0.8)		41 (10.0)
Without occupation	122 (65.6)	154 (68.8)		6 (1.5)
People living in the same residence	64 (34.4)	68 (30.4)	0.312	
Alone	131 (70.1)	156 (69.3)		2 (0.5)
1 - 3 people	56 (29.9)	69 (30.7)		276 (67.0)
≥ 4 people				132 (32.0)
Without occupation				2 (0.5)

*1 minimum wage in 2018: R$ 954 reais. Note: 1US dollar = R$ 3.88 reais

In Table 2, the clinical characteristics of the population that prevailed were people with a diagnosis time <10 years (68.5%), who never participated in an educational group (54%), non-insulin-dependent (91.3%), with adequate medical follow-up (52.8 %) and inadequate nursing (83.8%) and not practicing physical activity (75.5%).

Most people with insufficient knowledge had been diagnosed with DM2 for ten years or less, never participated in an educational group, underwent treatment without the use of insulin, underwent adequate periodic medical follow-up and inadequate nursing follow-up, and did not practice physical activity. Some clinical variables showed a statistically significant association with the level of knowledge, although the proportion of the level was inadequate in not participating in educational groups, using insulin, and not practicing physical activity.

**Table 2:** Clinical data of the study population, according to the level of knowledge about diabetes mellitus, 2018

Variables	Level of Knowledge	Level of Knowledge	***p*-value**	Total n (%)
Variables	Sufficient n (%)	Insufficient n (%)	***p*-value**	Total n (%)
				412 (100.0)
Time of diagnosis			0.360	
≤ 10 years	127 (70.1)	156 (72.9)		283 (68.5)
>10 years	54 (29,9)	58 (27.1)		112 (27.1)
Without occupation				17 (4.4)
Educational Group			0.001	
Participating/participated	97 (53.3)	80 (36.7)		177 (42.9)
Never participated	85 (46.7)	138 (63.3)		223 (54.0)
Without occupation				12 (3.1)
Treatment			0.047	
Not using insulin	165 (88.2)	211 (93.8)		376 (91.3)
Using insulin	22 (11.8)	14 (6.2)		36 (8.7)
Consultation with Doctor			0.463	
Adequate	95 (50.8)	123 (54.4)		218 (52.8)
Inadequate	92 (49.2)	102 (45.6)		194 (47.2)
Consultation with Nursing			0.326	
Adequate	34 (18.2)	32 (14.6)		66 (16.2)
Inadequate	153 (81.8)	193 (85.4)		346 (83.8)
Practicing Physical Activity			0.001	
Yes	50 (28.6)	34 (15.4)		84 (20.3)
No	125 (71.4)	187 (84.6)		312 (75.5)
Without occupation				16 (4.2)

From the gross and adjusted indicators of the final regression model, we could estimate an increased risk for insufficient knowledge in individuals who never participated in an educational group (OR=2.0 [95% CI 1.2 - 3.1]), aged 60 years old or older (OR = 2.2 [95% CI 1.3 - 3.6]), illiterate and with primary education (OR=8.4 [95% CI 2.2 - 31.7]) and with an income equal to or less than one minimum wage (OR=2.4 [95% CI 1.1 - 5.6])

**Table 3:** Odds ratio and 95% confidence interval of the final risk regression model in people with Type 2 Diabetes Mellitus for insufficient knowledge about the disease.

	Gross OR	Gross CI 95%	Adjusted OR	CI 95% Adjusted OR
Never participated in an Educational Group	1.9	1.2 - 3.0	2.0	1.2 - 3.1
Age ≥ 60 years old	2.1	1.2 - 3.5	2.2	1.3 - 3.6
Illiterate and primary education	7.5	1.9 - 28.6	8.4	2.2 - 31.7
Income ≤ 1 wage	2.1	0.9 - 5.2	2.4	1.1 - 5.6

## Discussion

In this study, the insufficient knowledge identified in 54.7% of the sample is related to a higher risk in individuals who never participated in an educational group, aged ≥ 60 years old, illiterate and primary school level and with an income less than or equal to 1 minimum wage. The prevalence of insufficient knowledge evidenced in this study is in line with national research.(13-18) A strong similarity was found with recent research carried out in Minas Gerais, where 56.1% had insufficient knowledge, associated with advanced age and low education level.(10) The relevance of having different professional conduct for the population aged ≥ 60 years old is necessary because it is a population with low adherence to drug therapy due to the lack of knowledge, complexity of the medication regimen, and relationship between the prescriber and the patient.(19)

Regarding education, similar data were evidenced in a Brazilian study that associated insufficient knowledge with low education. The participants had difficulty reading and understanding medical prescriptions, making adherence to treatment more difficult, and increasing health risks.(20) Low education is one of the causes of insufficient knowledge since most people with DM2 who had insufficient knowledge in this study had low education, and illiterate participants who had complete/incomplete primary education were eight times more likely to have insufficient knowledge. The population with diabetes monitored by PHC has a low level of education predominance.(17) Therefore, educational actions carried out in the context of PHC must be periodically evaluated and restructured to meet the needs of the population in each region.

Education is an important tool in the care of people with diabetes. Sufficient knowledge is possible through educational programs for self-care and care by a multi-professional team, seeking the empowerment of people with diabetes.(21) Scenarios that work with educational actions confirm the importance of education to increase the level of knowledge and consequently diabetes treatment. This study revealed that most never participated in an educational group. Thus, these people have 1.98 chances of having insufficient knowledge. Educational interventions are important for helping adherence to drug treatment, controlling glycated hemoglobin rates, improving self-care and quality of life, and especially decreasing the suffering of living with diabetes.(8) Also, people with sufficient knowledge tend to follow the therapeutic plan and avoid hyperglycemia and disease complications.(16)

Regarding the socioeconomic level, people with low family income are twice as likely to have insufficient knowledge.(16) In this study, most of the population with insufficient knowledge has an income below or equal to a minimum wage. People with diabetes who have low-income have difficulties to follow the therapeutic plan, the food changes increase food expenses because it is necessary to buy fresh food and periodic purchases.(22) Also, the free drugs distributed in the Units Health services are often unavailable, requiring travel to pharmacies that guarantee free prescription drugs.(23) The health of this population depends exclusively on SUS, which has weaknesses such as low quotas for periodic examinations and difficulty in referrals.

A significant association was also identified between living without a partner and insufficient knowledge. We observed that family support as a support network and support for coexistence is fundamental in adapting to the changes in habits required in the treatment of diabetes. The family must be evaluated and considered in the educational approach, where actions with an emphasis on autonomy and self-sufficiency should prevail. The association between insufficient knowledge and not performing physical activity may have been influenced by the predominance of low income and dependence on SUS in the study population. Within the scope of SUS in Brazil, the Heath Gym Program exists as a promotion strategy for bodily practices and physical activity. However, in the municipality of the study, this program has not yet been fully implemented.

Regarding the treatment, most people in the study did not use insulin, and this variable is associated with insufficient knowledge. A previous study showed that treatment with only diet or oral medication was presented by most of the population with insufficient knowledge and negative attitudes towards the disease.(10) The findings of this study are relevant in the context of public health since they point to the need for interventions under factors that can be easily modified, which would provide greater knowledge to individuals affected by DM. However, the transversal nature of this study and the lack of evaluation of other geographical realities could be pointed out as limitations of this study and, for this reason, direct new studies addressing the theme.

We conclude that the risk for insufficient knowledge is increased in individuals who have never participated in an educational group, aged 60 years old or over, illiterate and with primary education and with income equal to or less than the minimum wage. The level of knowledge found in this study evidenced the need for educational interventions that assist in the treatment of diabetes. This study recommends the implementation of multi-professional educational actions, considering the results presented to support the planning of actions.
